# Antimicrobial Potential of Secondary Metabolites Produced by Bacillus sp. and Their Gas Chromatography (GC)-Mass Spectrometry (MS) Analysis

**DOI:** 10.7759/cureus.70472

**Published:** 2024-09-29

**Authors:** Jesu Willson Ravi Sujitha, Dhivyashri Senthilkumar, Manivannan Nandhagopal

**Affiliations:** 1 Department of Microbiology, Saveetha Medical College and Hospitals, Saveetha Institute of Medical and Technical Sciences, Saveetha University, Chennai, IND

**Keywords:** antimicrobial activity, antioxidant activity, bacillus sp. extracellular metabolites, gc-ms analysis, halophiles

## Abstract

Aim

This study aims to determine the biological activity and explore the antimicrobial compounds produced* *by a halophilic bacterium, as well as the hemolytic, antioxidant, and antimicrobial properties of extracellular metabolites from *Bacillus *sp.

Methodology

The bacterial strain was obtained from the Department of Microbiology at Saveetha Medical College and Hospital, Chennai, India, specifically from the bio-control and microbial product laboratory (BCMPL). The genotype and phenotype of the isolate were characterized while the cultures were maintained in a nutrient broth medium supplemented with 8% sodium chloride (NaCl). The secondary metabolites were extracted using ethyl acetate and concentrated through open evaporation techniques after nine days of growth in the culture medium. Biological compatibility studies were conducted concurrently with the screening of the antimicrobial and antioxidant properties of the secondary metabolites. The chemical composition of the crude metabolites was analyzed using the gas chromatography (GC)-mass spectrometry (MS) technique.

Results

After phenotypic and genotypic analysis, the obtained potential halophilic bacterium from BCMPL was determined to be *Bacillus* sp. After the dark brown crude metabolites were extracted, the extracellular metabolites' antimicrobial activity against *Staphylococcus aureus *(*S. aureus*), *Escherichia coli *(*E. coli*)*,* and *Candida albicans* (*C. albicans*) extracellular metabolites was moderately inhibited. Furthermore, the metabolites exhibited a moderate level of hemolytic and antioxidant activity. The GC-MS method depicted the presence of 12 distinct metabolites, each with a distinct retention time.

Conclusion

To sum up, the halophilic bacteria that were obtained and identified as *Bacillus* species and their crude metabolites demonstrated noteworthy antioxidant and antimicrobial properties. Further investigation may be helpful in identifying possible compounds that *Bacillus* sp. produces.

## Introduction

Halophiles are microorganisms capable of thriving in high-salinity environments. It is a member of the eukaryotic, archaeal, and bacterial groups [1]. According to Sawale et al. [2], halophiles are present in numerous hypersaline conditions worldwide, including brines that naturally occur in arid, coastal, and deep-sea environments. According to how much salt they can withstand, halophiles are divided into four groups: mild (0.2-0.5 M), moderate (0.5-2.5 M), borderline extreme (2.5-4.0 M), and extreme (4.0-5.9 M) [3]. The ideal salt concentration for non-halophilic bacteria is less than 0.2 mol L-1 sodium chloride (NaCl). Both in situations with high salinity and low salt concentrations, halotolerant organisms are able to endure and proliferate. Because of remarkable cell variations such as salt-in strategy, enzyme adaptations, and compatible solute strategy, halophiles sustain high salt focus. Extremely high osmolarity can be harmful to cells because it results in water loss into the surrounding medium until osmotic equilibrium is achieved. Halophiles typically collect high solute foci inside the cytoplasm to prevent the loss of cell water in these circumstances [4]. Halophilic microorganisms have been used to produce numerous commercially significant enzymes, consisting of proteases, cellulases, lipases, amylases, and mannanases [5]. Halophiles can be used to create bioplastics, secondary metabolites, enzymes, and economically important solutes [6]. It has been demonstrated that halophilic organisms possess an extensive array of antibacterial activity against pathogens and produce a variety of antimicrobial compounds. Thus, these bioactive compounds can be directly applied therapeutically or utilized as lead compounds in the development of new medicines [7,8]. Furthermore, the bioactive metabolites that are separated from halophiles have a significant role in the manufacturing of biofuel as well as bioremediation [9]. The microorganisms recovered from the marine environment can be used for many biotechnological applications because the majority of halophiles can withstand higher pH, pressure, and temperatures [10]. Furthermore, according to Rohban et al. [11], distinct strains of halophilic bacteria produced lipases, proteases, amylases, inulinases, cellulases, xylanases, DNases, and pectinases, in that order. Biosurfactants with anticancer activity at varying concentrations are also produced by halophilic *Bacillus* sp. BS3 [2]. Thus, the purpose of this work is to determine the bioactivity of halophilic bacteria's intra- and extracellular metabolites that were collected from Paiyanoor, Tamil Nadu, India.

## Materials and methods

Halophilic isolate

The halophilic bacterium was obtained from the Department of Microbiology, Bio-control and Microbial Product Laboratory (BCMPL) at Saveetha Medical College and Hospital, Chennai, India, where the halophilic bacteria was originally isolated from Paiyanoor salt pan in Tamil Nadu. It was stored at 7°C in a nutrient agar slant medium containing 5% NaCl for further analysis.

Clinical pathogens

The antimicrobial activity was carried out using five distinct pathogenic bacteria obtained from the clinical microbiology lab, Department of Microbiology, Saveetha Medical College and Hospital, Chennai, namely, *Pseudomonas aeruginosa* (*P. aeruginosa*), *Candida albicans* (*C. albicans*), *Escherichia coli* (*E. coli*), *Enterococcus faecalis* (*E. faecalis*), and methicillin-resistant *Staphylococcus aureus* (MRSA). The pathogenic organisms were regularly cultivated and subcultured in Mueller-Hinton agar (MHA) medium (HIMEDIA M173-500G, HiMedia Laboratories Private Limited, Thane, Maharashtra) to maintain cell viability, while *C. albicans* was kept in Sabourad dextrose agar (SDA) medium.

Phenotypic and genotypic characteristics of the isolate

The obtained halophilic bacteria underwent morphological, physiological, and biochemical tests to determine their phenotypic characterization. According to the standard protocol suggested by Talaiekhozani [12], colonies on media plates were examined for color, form, and the outcomes of several biochemical tests, such as catalase and oxidase.

Bacterial nucleic acid Isolation

After being grown in a nutrient broth (NB) medium for 24 hours, the obtained halophilic bacteria were broken open by washing them in a phosphate-buffered solution (PBS). To facilitate the chemical lysis of the DNA, the lysis buffer was added. After adding the binding buffer to the lysate to denature the proteins and safeguard the DNA, the DNA was bound to silica. It was added to a spin column with a silica membrane; in the presence of a high concentration of salt, the DNA will bind to the membrane. To get rid of the impurities, the silica membrane was cleaned using a number of washing buffers. Water was utilized as the elution buffer to remove the DNA from the silica membrane, and agarose gel electrophoresis was used to examine the resultant DNA sample. Up to its next use, the extracted DNA was placed in storage at -20°C.

Amplification of nucleic acid

Using universal primers, the extracted DNA from the bacterial sample was amplified using polymerase chain reaction (PCR) to produce a copy of the 16S rRNA gene region. The conserved region was intensified with the help of primers 27F (5'-AGAGTTTGATCMTGGCTCAG-3') and 1492R (5'-GGTTACCTTGTTACGACTT-3'). Initial denaturation of the PCR is conducted at 95°C for five minutes. Thereafter, 35 cycles of 95°C for 30 seconds, 55-60°C for 30 seconds (annealing), and 72°C for 60 seconds (extension) are performed, with the final extension at 72°C for 10 minutes.

Sequencing and genomic examination of 16S rRNA

Sequencing

The refined PCR result was sent to Europeans Sequencing Center, Bangalore, a sequencing service provider, for Sanger sequencing. The FAST format sequencing data was obtained through the use of the European Molecular Biology Open Software Suite (EMBOSS) merger tool (Rice P, Bleasby A, and Ison J, http://emboss.open-bio.org/) which merged the forward and reverse primers (bioinformatics.nl). The nucleotides that were obtained underwent Basic Local Alignment Search Tool (BLAST) analysis through the National Center for Biotechnology Information (NCBI) Data Analysis Program. The resulting BLAST analysis data was downloaded as a FASTA file. The neighbor-joining strategy was utilized to surmise the evolutionary history [13]. The bootstrap agreement tree obtained from 1,000 replicates depicted the evolutionary history of the taxa under examination [14]. Divisions associated with replicated partitions were compressed inferior to 50% of bootstrap replicates. Tamura et al. [15] employed the Maximum Composite Likelihood technique to calculate the evolutionary distances. This required looking at 58 different nucleotide sequences. Utilizing the pairwise cancellation option, ambiguous positions were eliminated for each sequence pair, leaving the last dataset with 1,632 positions overall. Molecular Evolutionary Genetics Analysis version 11 (MEGA 11) (Tamura, Stecher, and Kumar, 2021, https://www.megasoftware.net/) was utilized to complete the evolutionary investigations [16].

Production and extraction of secondary metabolites

Microbial metabolites were produced in a nutrient-rich medium containing 8% sodium chloride. The bacterial culture obtained was cultivated for nine days in 250 milliliters of NB medium at a temperature of 35 °C ± 2 until a change in the color of the growth medium was observed. Centrifugation was used to gather the cell-free supernatant after growth, and filtration came next. After adding half of ethyl acetate to the obtained culture filtrate and thoroughly mixing it inside a separating funnel, the ethyl acetate was isolated and collected in a different conical flask. This process allowed for liquid-liquid extraction. The extracts made of ethyl acetate were condensed and kept for later research.

Antimicrobial assay: well diffusion method

The well diffusion assay was used to assess the antimicrobial activity of the mass-produced secondary metabolites against five distinct microorganisms: MRSA,* E. faecalis*,* E. coli*, *P. aeruginosa*, and *C. albicans*. Sterile Mueller-Hinton broth (MHB) was used to develop the microorganisms as mother inoculum, and their optical density was adjusted to 0.4 at 600 nm. The microorganisms were then swabbed onto sterile Mueller-Hinton agar medium plates. Using a cork borer, wells were created in the agar and filled with 100 µL of the crude metabolites. The plates were incubated for 16 hours at 37°C, and the inhibition zones were measured using a zone scale. The experiment was carried out in triplicate.

Minimum inhibitory concentration (MIC)

As per the Clinical and Laboratory Standards Institute (CLSI) and European Committee on Antimicrobial Susceptibility Testing (EUCAST) guidelines, the MICs were evaluated as previously conducted by Nandhagopal et al. [17]. The micro-titer test plates were loaded up using differing concentrations of crude metabolites (512-1 mg/L), and everything except the negative control well-received 5 μL of human pathogens for the positive controls. Amoxicillin for Gram-positive pathogens, levofloxacin for Gram-negative pathogens, and fluconazole for *Candida* sp. were used. The plates were incubated at 37°C for an entire day. Following the incubation period, 10 μL of recently pre-prepared3-(4,5-dimethylthiazol-2-yl)- 2,5-diphenyl tetrazolium bromide (MTT) (5 mg/mL) was added. A 100% dimethyl sulfoxide (DMSO) was then added, and the optical density (OD) was estimated in an enzyme-linked immunosorbent assay (ELISA) reader at 595 nm to decide the level of cell death. The outcomes were all recorded.

Antioxidant activity of secondary metabolties from halophilic bacterium

The 2,2-diphenyl-1-picrylhydrazyl (DPPH) radical scavenging method to ascertain the antioxidative capacity of crude metabolites. First, DPPH was added to undiluted metabolites in concentrations within the range from 1 mg/L to 512. After that, the mixture was allowed to sit at room temperature for 30 minutes in the dark. One milliliter of methanol and two milliliters of DPPH were used as a negative control, and only methanol solution was used as a positive control. At 517 nm, absorbance was measured in order to determine the percentage scavenging activity (% radical scavenging activity (RSA)) of DPPH radicals.

$\text{Antioxidant Activity} (\%) = \frac{\text{Absorbance of Control} - \text{Absorbance of Sample}}{\text{Absorbance of Control}} \times 100$

Hemolytic activity

Human blood cells were used to test the biological compatibility of secondary metabolites from *Bacillus *sp. the recently drawn human blood cells from the participants, followed by three PBS washes. The PBS was used as the negative control, and 1% Triton X-100 (HiMedia Laboratories Private Limited) was the positive control. The various concentrations of secondary metabolites (100, 50, 25, 12.5, 6.25, 3.12, 1.56, 0.78, μg/ml) were diluted in 800 µL of PBS solution, and 200 μL of blood sample was added to each microcentrifuge tube separately. Sterilized microfilter plates were used after the tubes were centrifuged at 5000 rpm for seven minutes and incubated for an hour at 37 °C. At 570 nm, absorbance measurements were made, and the amount of hemoglobin released was computed.

$\text{Hemolysis} (\%) = \frac{\text{Absorbance of Sample} - \text{Absorbance of Control}}{\text{Absorbance of Positive Control} - \text{Absorbance of Control}} \times 100$

These investigations were done twice, and results are expressed by mean ± standard deviation (±SD).

Gas chromatography (GC)-mass spectrometry (MS) analysis of secondary metabolites produced by halophilic bacteria

To analyze the molecules present in *Bacillus *sp.'s secondary metabolites were examined using the GC Ultra and DSQ II model mass spectrometers (Thermo Fischer Scientific, Waltham, MA). The temperature of the injector port was set to 250°C, the connection point temperature to 250°C, the source temperature to 200 °C, and the engine vacuum strain to 40 psi on the instrument. The oven's temperature could be adjusted to 70°C for two minutes, 150°C at 8°C/min, and as high as 260°C at 10°C/min. DB-35ms (Agilent Technologies, Inc., Santa Clara, CA) a non-polar section with dimensions of 0.25 μm inner diameter (ID) x 0.25 mm outer diameter (OD) was used. Helium was the carrier gas, and it was used at a rate of 1 ml/min. The mass spectrometer was programmed to search for fragments ranging in mass from 650 Da to 50 Da. A pre-filter was included in the MS to eliminate neutral particles, and the ionization energy was -70 eV. The outcomes were analyzed with the library's reference data.

Statistical analysis

Every experiment was conducted twice or three times, and the mean ± standard deviations (SD) were used to express the results. GraphPad Prism software (GraphPad Software, La Jolla, CA).

## Results

Morphological and phenotypic characterization of halophilic bacteria

**Figure 1:**
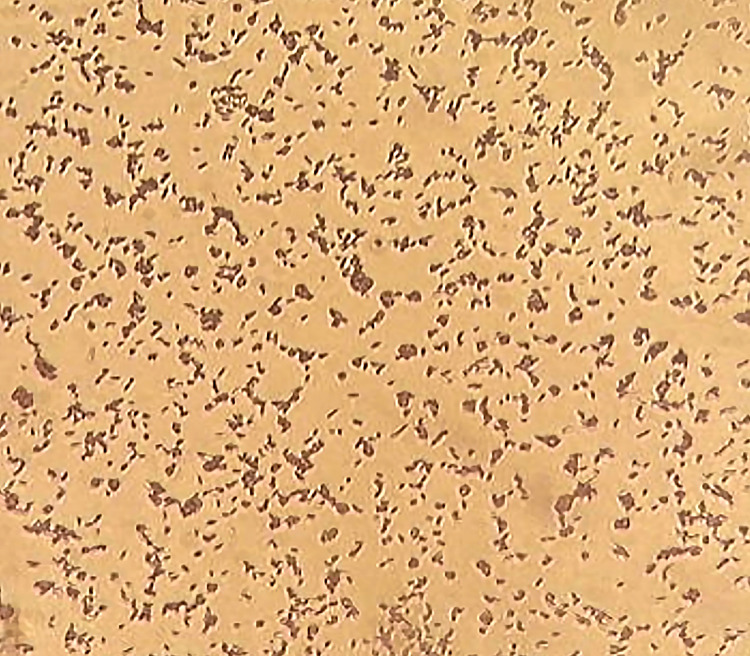
Microscopic observation of halophilic bacteria

In nutrient agar medium with 8% NaCl, the halophilic bacteria's morphology revealed light yellow, round-shaped, flat, and translucent colonies. Following Gram's staining, Gram-positive bacilli were seen in light microscopy (Figure 1).

Numerous biochemical tests yielded positive results for catalase and indole and negative results for methyl red, Voges-Proskauer, citrate utilization, urease, and nitrate reduction.

Molecular identification of bacterial isolates

**Figure 2:**
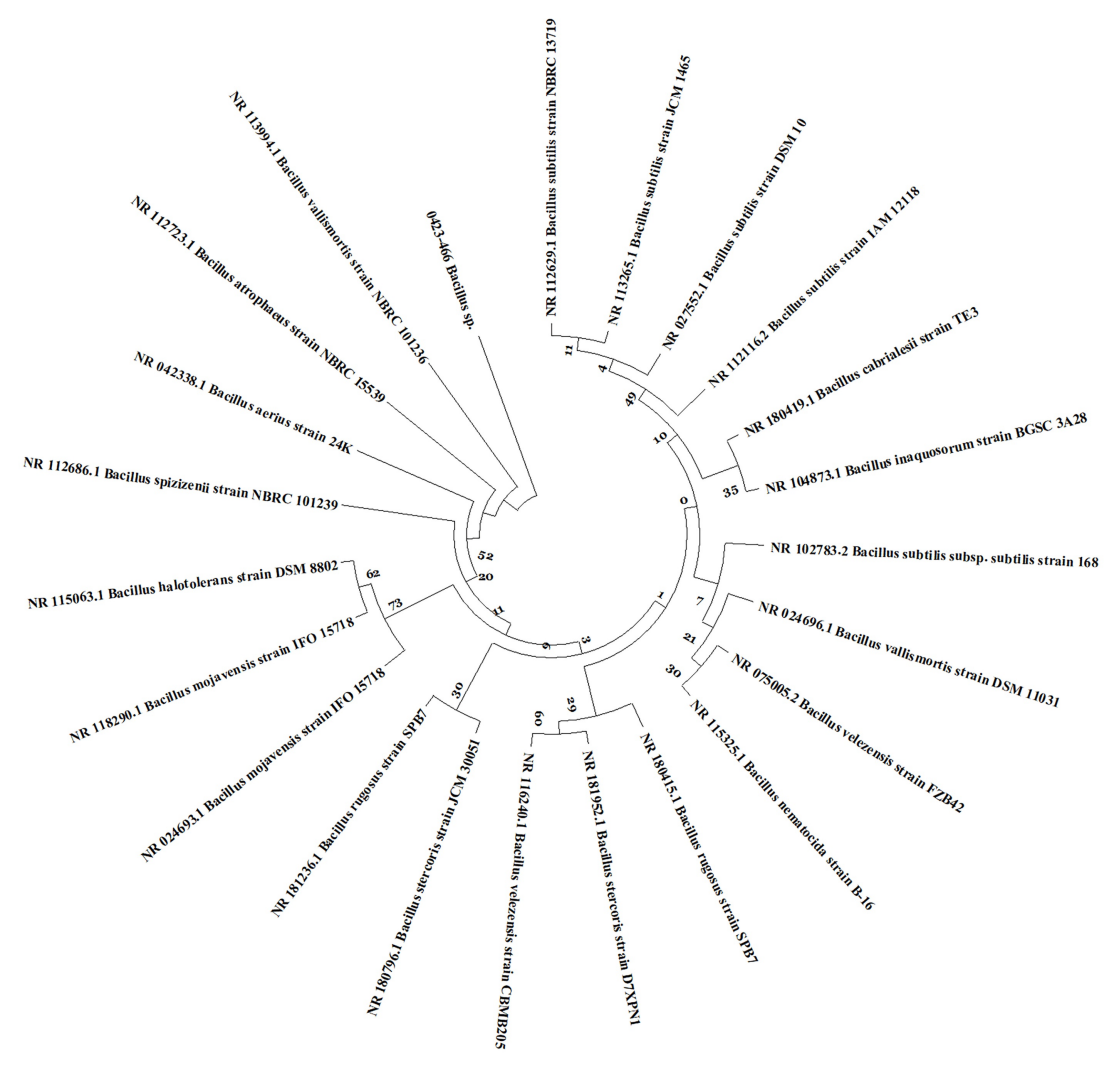
Phylogenetic tree construction of halophilic bacteria The image was created by the authors using the software Molecular Evolutionary Genetics Analysis, version 11 (MEGA 11) developed by Tamura, Stecher, and Kumar, 2021 (https://www.megasoftware.net/).

Using the Sanger method, the genomic DNA of a potent bacterial strain was separated, and PCR products were produced for identification via 16S rRNA gene sequencing. Using the BLAST 2.0 program (National Library of Medicine,Bethesda, MD) to identify the isolate of potent bacterium, the DNA sequences were compared to those found in the GenBank NCBI database. Following the BLAST analysis, we discovered that the halophilic organism under investigation shared 97% similarities with the *Bacillus stercoris* strain D7XPN1,* Bacillus velezensis* strain CBMB205, *Bacillus rugosus* strain SPB7, *Bacillus vallismortis* strain NBRC 101236, and *Bacillus spizizenii* strain NBRC 101239. Further, it resembled the *Bacillus mojavensis* strain IFO 15718 (96%), *Bacillus atrophaeus* strain NBRC 15539, *Bacillus aerius* strain 24K, and *Bacillus haikouensis* strain C-89 (95%) (Figure 2).

Antimicrobial activity of secondary metabolites

**Figure 3:**
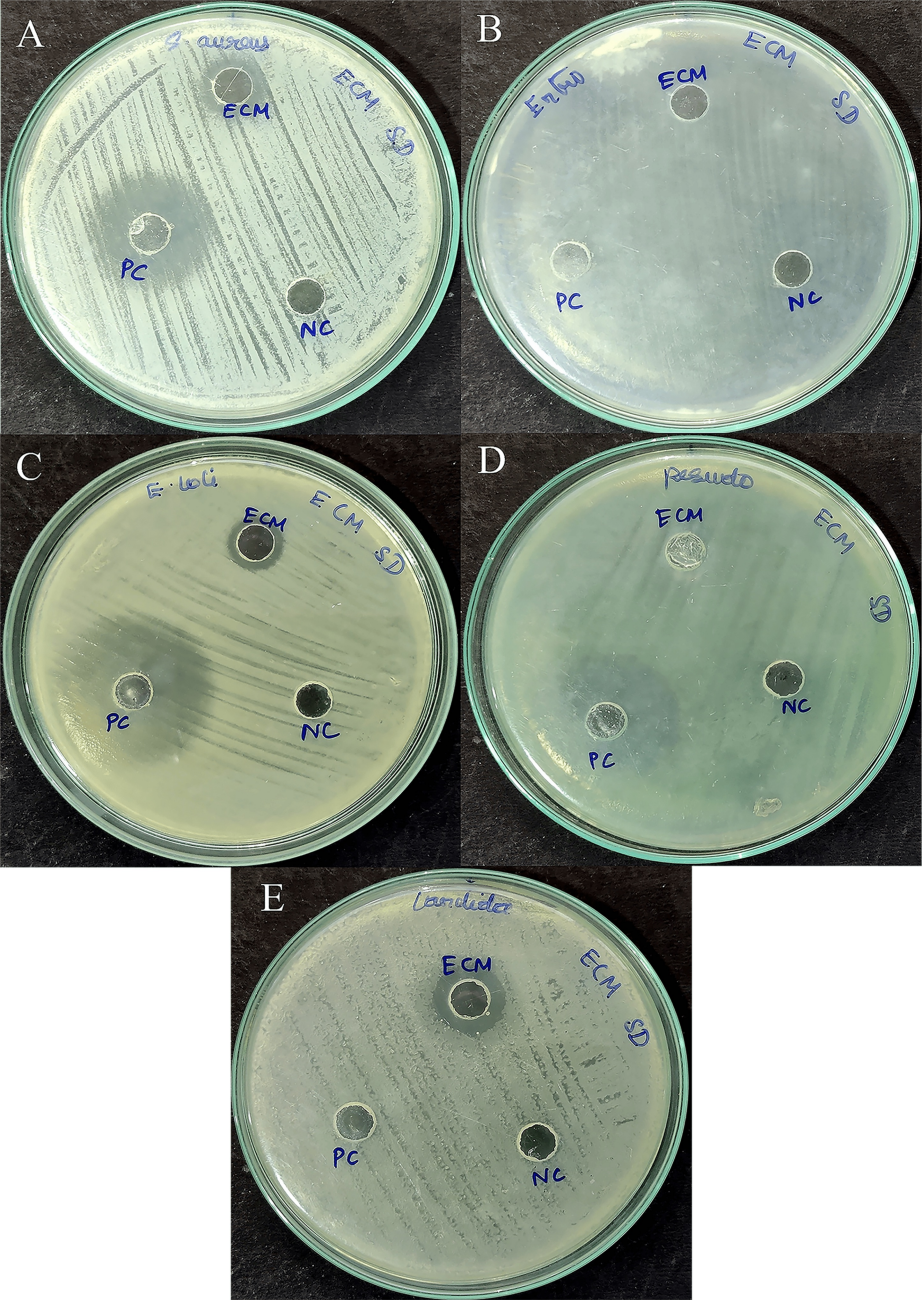
Antimicrobial activity of secondary metabolites from Bacillus sp. A: Methicillin-resistant *Staphylococcus aureus* (MRSA); B: *Enterococcus faecalis; *C: *Escherichia coli; *D: *Pseudomonas aeruginosa*​​​​​​​*; *E: *Candida albicans*​​​​​​​*; *ECM: extracellular metabolites; NC: negative control; PC: positive control

Extracellular metabolites' antimicrobial potential was demonstrated by a zone of inhibition measuring 12 mm against *Staphylococcus aureus* (*S. aureus*), 11 mm against *E. coli* and *E. faecalis*, and 13 mm against *C. albicans*. However, no antimicrobial activity was observed against *P. aeruginosa* or *E. faecalis* (Figure 3 and Table 1).

**Table 1:** Antimicrobial activity of extra cellular metabolites produced by Bacillus sp. ECM: extracellular metabolites; NC: negative control; PC: positive control

Sample	Zone of Inhibition in mm				
	Gram-positive bacterium		Gram-negative bacterium		Fungal pathogen
	Staphylococcus aureus	Enterococcus faecalis	Escherichia coli	Pseudomonas aeruginosa	Candida albicans
ECM	12	11	11	-	13
NC	-	-	-	-	-
PC	24	15	29	27	-

The MIC of extracellular metabolites produced by *Bacillus* sp.

The* Bacillus *sp. microbial metabolites' MIC was found to be effective against the five clinical pathogens. The extracellular metabolites' MIC against *S. aureus* was 128 mg/L, meaning that the metabolites could stop the growth of bacteria at concentrations of 128 mg/L for* S. aureus*, *C. albicans*, and *E. faecalis*, and 256 mg/L for *E. coli* and *P. aeruginosa* (Table 2).

**Table 2:** The MIC of ECM produced by Bacillus sp. MIC: minimum inhibitory control; ECM: extracellular metabolites; PC: positive control

Metabolites	MIC (mg/L)									
	Gram-positive bacterium				Gram-negative bacterium				Fungal pathogen	
	Staphylococcus aureus		Enterococcus faecalis		Escherichia coli		Pseudomonas aeruginosa		Candida albicans	
	ECM	PC	ECM	PC	ECM	PC	ECM	PC	ECM	PC
MIC value	128	2	256	128	512	1	512	2	128	ND

Antioxidant activity of microbial metabolites from *Bacillus* sp.

**Figure 4:**
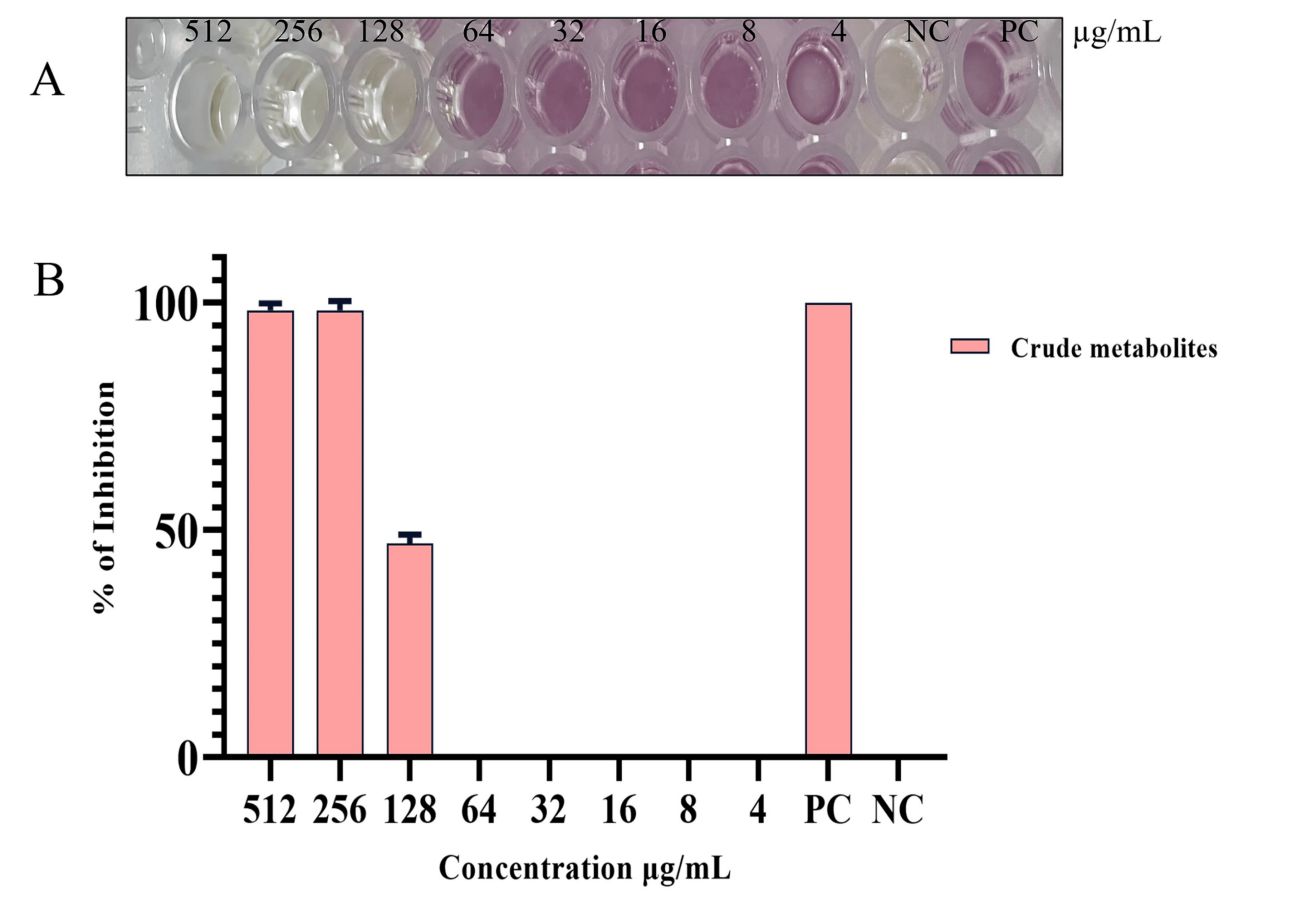
Antioxidant activity of extracellular metabolites produced by Bacillus sp. A: antioxidant activity; B: percentage of antioxidant activity NC: negative control; PC: positive control

Lipids, extracellular matrix, and intracellular antioxidant activity were measured. The intracellular metabolite was found to be able to decrease at concentrations of 512 µg/mL, 256 µg/mL, and 128 µg/mL. On the other hand, at 512 and 256 µg/mL, the extracellular metabolite was able to lower the free radicals. The lipid extract, however, did not exhibit any antioxidant qualities (Figure 4).

Hemolytic activity of the microbial metabolites from *Bacillus* sp.

**Figure 5:**
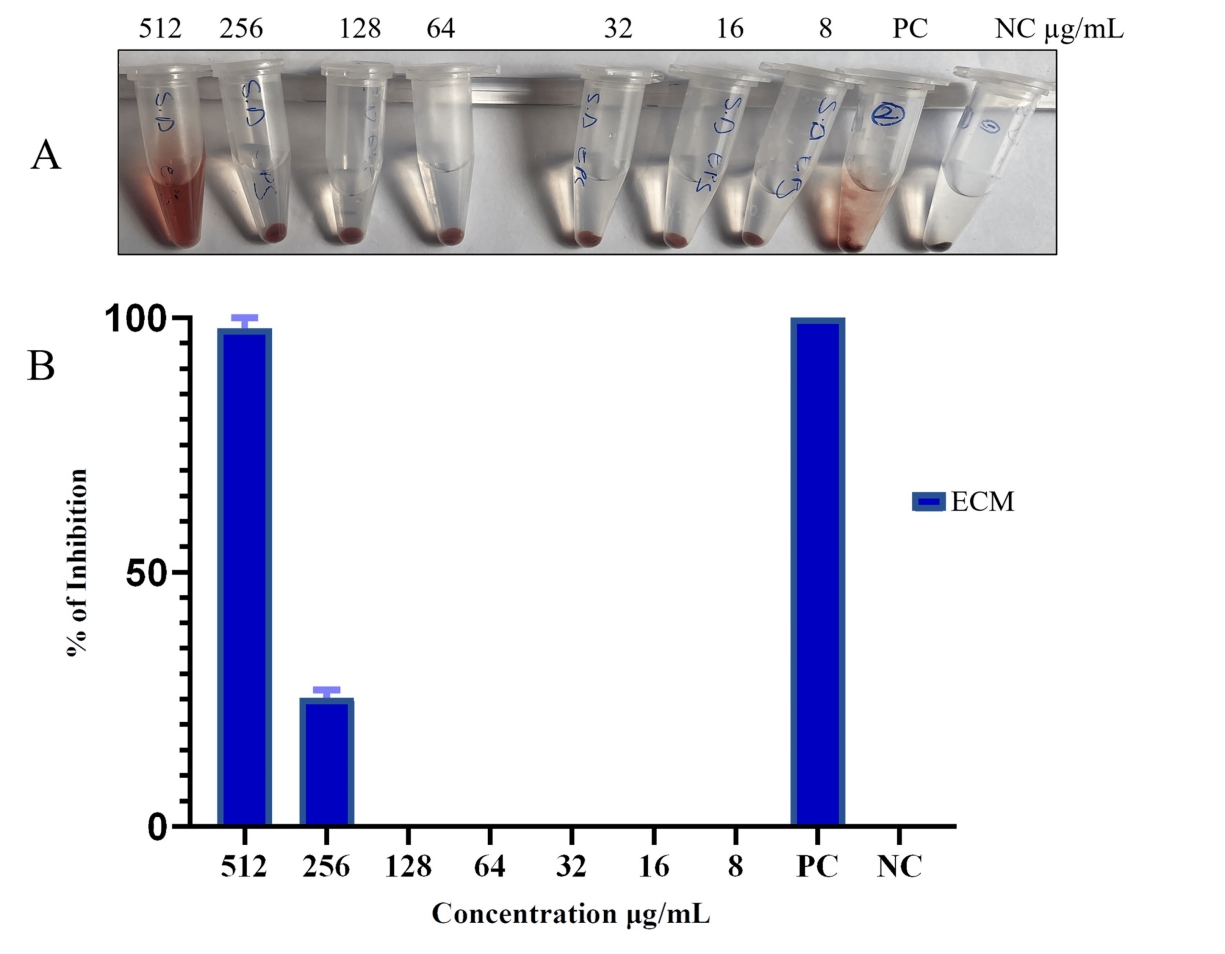
Hemolytic activity of extracellular metabolites produced by Bacillus sp. A: hemolytic activity; B: percentage of hemolytic activity ECM: extracellular metabolites; PC: positive control; NC: negative control

Notable outcomes were obtained from the hemolytic activity of the extracellular metabolites generated by *Bacillus *sp. At 512 µg/mL, there was 99% lysis, indicating high hemolytic activity. Nevertheless, at 256 µg/mL, hemolytic activity dropped to 36%, suggesting less hemolytic activity at lower concentrations. Hemolytic activity was absent below a concentration of 128 µg/mL indicating the critical function of concentration on the ECM (Figure 5).

The GC-MS analysis of extracellular metabolites produced by *Bacillus* sp.

**Figure 6:**
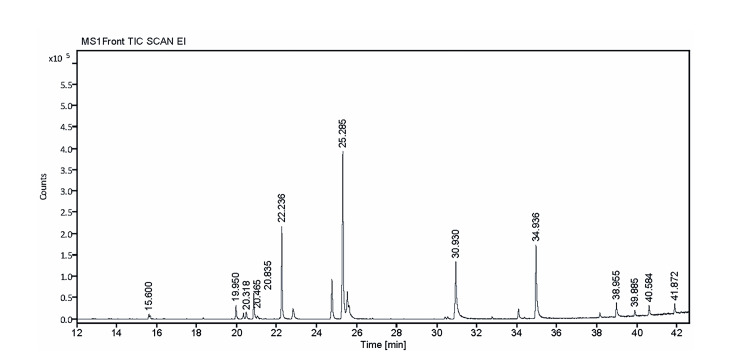
The gas chromatography-mass spectrometry chromatograme of extra cellular metabolites produced by Bacillus sp.

The secondary metabolite analysis by GC-MS of *Bacillus* sp. depicts the existence of 12 distinct compounds in the crude metabolites (Table 3), of which four were determined to be major based on their area percentage and probability percentage, as follows: 1. With an area percentage of 40.19, pyrrolo[1,2-a]pyrazine-1,4-dione, hexahydro-3-(2-methylpropyl)- showed the largest value. Additionally, the probability of 95.2 was extremely high. The metabolite cyclo(L-prolyl-L-valine) exhibited noteworthy prominence as well, with a probability of 93.72% and an area percentage of 16.41. The metabolite 2,5-piperazinedione, 3,6-bis(2-methylpropyl)- had an area percentage of 11.36 with a probability percentage of 71.66, and the metabolite pyrrolo[1,2-a]pyrazine-1,4-dione, hexahydro-3-(phenylmethyl)- had a significant area percentage of 12.20 with a high probability percentage of 84.4. Based on the provided area percentage and probability percentage, each of these metabolites indicates a high likelihood of presence (Figure 6 and Table 3).

**Table 3:** Gas chromatography-mass spectrometry analysis of crude metabolites form Bacillus sp.

Peak	Retention time	Area %	Name	Probability %	Formula	Molecular weight
1	15.60	0.67	Acetamide, N-(2-phenylethyl)-	67.14	C10H13NO	163.22
2	19.950	2.51	1,4-diazabicyclo[4.3.0]nonan-2,5-dione, 3-methyl	94.87	C8H12N2O2	168.19
3	20.318	0.88	(3S,6S)-3-Butyl-6-methylpiperazine-2,5-dione	50.02	C9H16H2O2	184.12
4	20.465	1.36	dl-Alanyl-l-leucine	1.36	C19H18N2O3	202.25
5	20.835	5.06	Pyrrolo[1,2-a]pyrazine-1,4-dione, hexahydro-	72.82	C7H10N2O2	154.17
6	22.236	16.41	Cyclo(L-prolyl-L-valine)	16.41	C10H16N2O2	196.12
7	25.285	40.19	Pyrrolo[1,2-a]pyrazine-1,4-dione, hexahydro-3-(2-methylpropyl)-	95%	C11H18N2O2	210.27
8	30.930	11.36	2,5-Piperazinedione, 3,6-bis(2-methylpropyl)-	71.66	C12H22N202	226.17
9	34.936	12.20	Pyrrolo[1,2-a]pyrazine-1,4-dione, hexahydro-3-(phenylmethyl)-	84.4	C14H16N2O2	224.29
10	38.955	3.62	Cyclo-(l-leucyl-l-phenylalanyl)	32.7	C15H20N2O2	260.33
11	40.584	2.38	Glafenin	88.32	C19H17ClN2O4	372.80
12	41.872	2.18	3,3'-Diindolylmethane	65.44	C17H14N2	246.12

## Discussion

The marine environment is home to the planet's largest ecosystem. Within the world's top 12 bio-variety districts, India has a coastline spanning approximately 7,500 kilometers. Tamil Nadu, which stretches across the Bay of Bengal, the Indian Ocean, and the Arabian Sea, makes up about 15% of all of India's coastline. About 1,076 kilometers make up this [18,19]. Nearly every kind of intertidal zone, saltwater and brackish lagoons, estuaries, coastal marshes, rocky and sandy seas with varying degrees of exposure, and distinctive profiles can all be found in the ocean [19]. Halophilic bacteria are a special kind of bacteria that can survive in extremely salinized conditions. These conditions can be found in salt lakes, salt pans, and the deep ocean. Most other organisms would perish in high salt concentrations, but these bacteria have evolved to thrive there [20-22]. They have altered the structure of their proteins to enable them to function in high salinity conditions. High salinity environments are necessary for the biological functions of halophiles, including DNA replication, ribosome activity, cell membrane function, and metabolic enzyme performance [23,24].

The halophilic bacteria that we isolated and identified as *Bacillus *sp. from a salt pan in Paiyanoor, Tamil Nadu, India, are the subject of this study. Despite being ignored or less well-known, halophilic bacteria have a variety of functions and potential applications in real life. A Gram-positive and spore-forming bacterium, *Bacillus *sp., is commonly reported to be halophilic or halotolerant [25,26]. According to Yin et al. [27], *Bacillus *species are frequently found in high concentrations in saline environments like salt lakes, salt mines, and pans. These bacteria use a mechanism called the "salt-in strategy" to survive in high osmotic pressure environments, such as high salt concentrations. This strategy safeguards the bacterial function and metabolisms [27,28]. Additionally, we discovered in this study that *Bacillus *sp. exhibits strong similarities to the Lewis Bac 18 strain of *Bacillus vallismortis*. Gram-positive *Bacillus *​​​​​​*vallismortis* is a rod-shaped bacterium that is a member of the *Bacillus *genus. Like other bacteria in the *Bacillus *genus, *Bacillus* ​​​​​​​*vallismortis* can produce endospores, which are robust structures that can withstand environmental stress [29]. *Bacillus *sp. has been shown to be useful in bioremediation, particularly in the treatment of pyrene-contaminated environments [30]. *Bacillus vallismortis *JB201 possesses the ability to produce a lipopeptide biosurfactant that exhibited significant antimicrobial characteristics. Chakraborty et al. [31] tested these compounds against a variety of common human pathogens and found that they were effective against *Salmonella typhi*, *E. coli*, and *Klebsiella pneumonia* (*K. pneumoniae*). According to Zhao et al. [29], the study discovered that *Bacillus vallismortis* ZZ185, which was isolated from the plant Broadleaf Holly, exhibited potent in vitro antifungal activity against *A. alternata* and F*. graminearum*, two phytopathogens. These earlier studies demonstrate the efficacy of *Bacillus* *vallismortis*. In addition, other *Bacillus *species exhibit strong potential against a range of pathogens. As per the findings of Jamil et al. (2020) [32], the halophilic strain SP 52, along with its crude metabolites, demonstrated a zone of inhibition against *E. coli*. It has been demonstrated that these halophilic strains have no effect on *P. aeruginosa* or *K. pneumoniae*. In contrast, the halophilic bacteria's extracellular metabolites in our investigation demonstrated antimicrobial interactions against *S. aureus*, *E. coli*, and *C. albicans*. There was no antibacterial activity demonstrated by *P. aeruginosa*. The halophilic bacteria (*Bacillus *sp.) produce secondary metabolites with moderate antioxidant activity and high biocompatibility with human blood cells when their concentration is below 128 µg/mL. Twelve distinct molecules were detected in the GC-MS analysis, of which four were determined to be major molecules and may be responsible for the biological activity. To identify the molecules in charge of the biological activity, more research must be done.

Limitations of the study

While this study highlights the promising antimicrobial and antioxidant potential of metabolites from a single *Bacillus *sp., it's crucial to acknowledge certain limitations. The study primarily focuses on a single *Bacillus *sp., and exploring a wider diversity of halophilic bacteria could reveal strains with even more potent bioactivities. While the study notes moderate antimicrobial activity, further research using quantitative assays is needed to determine precise minimum inhibitory concentrations against a broader range of clinically relevant pathogens. Additionally, the study relies on crude extracts, and isolating and characterizing individual bioactive compounds is essential to understanding their specific mechanisms of action and therapeutic potential. Furthermore, the observed hemolytic activity, even at moderate levels, necessitates careful consideration of potential toxicity and warrants further investigation into safer derivatives or delivery methods. Finally, the study's in vitro nature underscores the need for in vivo studies to validate these findings and assess the true therapeutic potential of these metabolites.

## Conclusions

In conclusion, *Bacillus* sp.'s secondary metabolites have antimicrobial activity that shows promise for use in biomedical applications, particularly in the control of multidrug-resistant human pathogens. These bacteria's adaptability and resilience, which may result in the discovery of strong bioactive compounds, are demonstrated by their ability to flourish in highly salinized environments. These bacterias' antimicrobial compounds have the potential to be created as novel antimicrobial agents. These results may facilitate the development of novel antimicrobial compounds and efficient biocontrol agents through additional research. *Bacillus *species and their secondary metabolites thus offer a fascinating and promising field for further study and real-world applications.
